# EEG Oscillation Evidences of Enhanced Susceptibility to Emotional Stimuli during Adolescence

**DOI:** 10.3389/fpsyg.2016.00616

**Published:** 2016-05-18

**Authors:** Xianxin Meng, Wenwen Liu, Ling Zhang, Xiang Li, Bo Yao, Xinsheng Ding, JiaJin Yuan, Jiemin Yang

**Affiliations:** ^1^Faculty of Psychology, Southwest UniversityChongqing, China; ^2^School of Education, Nanyang Normal UniversityNanyang, China; ^3^Sichuan Information Technology CollegeGuangyuan, China

**Keywords:** adolescence, susceptibility, negative stimuli, time-frequency analysis (TFA), event-related synchronization (ERS), event-related desynchronization (ERD)

## Abstract

**Background:** Our recent event-related potential (ERP) study showed that adolescents are more emotionally sensitive to negative events compared to adults, regardless of the valence strength of the events. The current work aimed to confirm this age-related difference in response to emotional stimuli of diverse intensities by examining Electroencephalography (EEG) oscillatory power in time-frequency analysis.

**Methods:** Time-frequency analyses were performed on the EEG data recorded for highly negative (HN), moderately negative (MN) and Neutral pictures in 20 adolescents and 20 adults during a covert emotional task. The results showed a significant age by emotion interaction effect in the theta and beta oscillatory power during the 500–600 ms post stimulus.

**Results:** Adolescents showed significantly less pronounced theta synchronization (ERS, 5.5–7.5 Hz) for HN stimuli, and larger beta desynchronization (ERD; 18–20 Hz) for both HN and MN stimuli, in comparison with neutral stimuli. By contrast, adults exhibited no significant emotion effects in theta and beta frequency bands. In addition, the analysis of the alpha spectral power (10.5–12 Hz; 850–950 ms) showed a main effect of emotion, while the emotion by age interaction was not significant. Irrespective of adolescents or adults, HN and MN stimuli elicited enhanced alpha suppression compared to Neutral stimuli, while the alpha power was similar across HN and MN conditions.

**Conclusions:** These results confirmed prior findings that adolescents are more sensitive to emotionally negative stimuli compared to adults, regardless of emotion intensity, possibly due to the developing prefrontal control system during adolescence.

## Introduction

Adolescence is a developmental period characterized by mood instability, such as more engagement in risky behaviors and persistent negative and labile mood states (Spear, 2000; Ernst et al., 2006; Dahl and Gunnar, 2009; Somerville et al., 2010). It has been reported that the function of prefrontal cortex is immature (Lewis et al., 2006; Blakemore, 2008a,b; Hare et al., 2008), and that prefrontal modulation of subcortical inputs is still developing during adolescence (Lewis et al., 2006; Hare et al., 2008). Also, the immature orbital and medial prefrontal cortices are implicated in aggression and socially inappropriate behaviors in adolescents (Lewis et al., 2006; Carretié et al., 2009). Many neuroimaging studies unraveled that the neural circuits for emotion processing still undergo development during adolescence (Luna et al., 2001; Passarotti et al., 2009; Wong et al., 2009). For example, Passarotti et al. (2009) used fMRI technique to compare neural bases between adolescents and adults during facial emotion processing. Adolescents, compared with adults, showed less activation in right ventrolateral prefrontal cortex (VLPFC) and greater activation in paralimbic regions during a non-emotional task, suggesting that adolescents have increased emotional responding and immature prefrontal modulation of subcortical emotional inputs compared to adults. Using a standard/deviant distinction task and ERP method, our recent study manipulated the intensity of negative stimuli, and observed that adolescents showed enhanced emotion effect to negative stimuli compared to adults in attentional indexes (like, frontal P200, and N200), regardless of the emotional intensity of the stimuli.

Notably, since the ERP averaging technique filters out the contributions of those induced neural activities that are not phase-locked to the time-locking events by means of phase cancelation (Yuan et al., 2014; Chen et al., 2015), the ERP does not reflect the event-related brain dynamics comprehensively. Time-frequency analysis (TFA) of event-related spectral perturbation (ERSP) before epoch averaging can provide complementary information on neural processing dynamics that is distinctive from traditional phase-locked ERP method (Chen et al., 2015). As described in our prior study (Yuan et al., 2015), emotionally evocative scenes elicit more of direct emotion responding, while faces elicit the processing of emotion recognition (Herba and Phillips, 2004; Proverbio et al., 2009). On the other hand, an experimental design that does not require subjects to evaluate emotion may allow emotional responses in the laboratory setting to more closely resemble those in natural situations. Thus, Electroencephalography (EEG) data induced by evocative pictures during an implicit emotional task may be an analog to the emotional activity in real-life settings.

Based on these considerations, the current study applied TFA to the EEG data described in our prior ERP study (Yuan et al., 2015), to examine how brain responses to emotional pictures of varying intensities, as indexed by neural oscillations, vary between adolescents and adults. Neural oscillation dynamics can be assessed by the ERSP, and quantified with power change within a frequency band: the relative event-related EEG power increase (or decrease) in a frequency band was termed as event-related synchronization (ERS) (or event-related desynchronization, ERD) (Pfurtscheller, 1999; Passarotti et al., 2009; Chen et al., 2015). Indeed, ERSP measures have been widely used in emotion studies in recent years (Klimesch et al., 2007; Knyazev, 2007; Chen et al., 2015). Several studies observed emotional modulation of beta ERD (Chen et al., 2015) and alpha ERD (Hirata et al., 2007; Klimesch et al., 2007; Knyazev, 2007; Mu et al., 2008), and theta ERS (Balconi and Lucchiari, 2006; Balconi and Pozzoli, 2009; Gärtner and Bajbouj, 2014).

Several frequency oscillations have been reported to be related to emotional processing (Aftanas et al., 2001; Knyazev, 2007; Balconi and Pozzoli, 2009). It has been observed that both theta ERS (Aftanas et al., 2001, 2004; Balconi and Lucchiari, 2006; Balconi and Pozzoli, 2009) and beta ERD (Chen et al., 2015) are related to orienting attention. As adolescents are known for enhanced attention orienting to negative stimuli (Yuan et al., 2015), and for immature prefrontal function of regulating subcortical emotional inputs (Passarotti et al., 2009; Yuan et al., 2015), it is likely to observe larger emotion effect in theta ERS and beta ERD in adolescents compared to adults. Moreover, alpha ERD is involved in the regulation or inhibition of negative emotion (Knyazev, 2007; Mu et al., 2008). As the distracting task (i.e., standard/deviant distinction by pressing different keys) is easy and the behavioral performances of both samples reached a ceiling effect (Yuan et al., 2015), it is reasonable to anticipate that the two samples may recruit a similar extent of inhibitory processing of task-irrelevant emotional information. Thus, we hypothesized that adolescents and adults may show similar emotion effect in alpha ERD.

## Materials and Methods

### Participants

As paid volunteers, 20 adolescents aged in 13–14 years (10 boys, *M* = 13.70, *SE* = 0.11) and 20 adults in the age range 20–22 years (10 men, *M* = 20.75 years, *SE* = 0.47) were included in this study. All the subjects were free of brain illness history, psychiatric disorders or medications. Additionally, both samples were right-handed, with normal or corrected to normal eyesight. Also, the subjects recruited for this study were emotionally healthy, free of anxious /depressive disturbances, as we screened for possible affective disturbances prior to the experiment. Adolescents were debriefed with respect to emotional stability in the past 2 weeks, by reporting whether they had the following symptoms by a yes/no fashion: irritable, uneasy, anxious, and depression. Adolescents and their parents who reported no symptoms in these domains were recruited for the experiment. Adult subjects were screened for emotional instability by NEO-FFI-neuroticism assessment. The adult subjects recruited for EEG recording were emotionally stable, as evidenced by the significant below-threshold (0) scoring in neuroticism assessment [*M* = –26.45; *SE* = 4.2; *p* < 0.001]. Adolescents were sampled by administrating the test of the Project on Human Development in Chicago Neighborhoods (PHDCNs): Pubertal Development Scale (PDS, Wave 3) (Petersen et al., 1988; Earls et al., 2002). The pubertal status of adolescents recruited for this study was determined by the following three criteria: (1), the scoring in each item of the PDS measure ranged from 2 to 4; (2), nobody reported completion of physical development (total score <20); and (3), girls reported menarche and obvious breast growth, and boys reported deepening of voice and obvious facial hair growth (equivalent to Tanner Stage 3–4). Each adolescent had the prominent features of human puberty, according to their scores in the puberty measure (12.90 ± 2.26:M ± SD). Reanalysis of the data were approved by the IRB of the School of Psychology (the number of IRB: 201601). Written informed consent was obtained prior to the study from all adults, and from parents/legal guardians to adolescents. The experimental procedure was in accordance with the ethical principles of the 1964 Declaration of Helsinki (World Medical Organization, 1996).

#### Experimental Task

**FIGURE 1:**
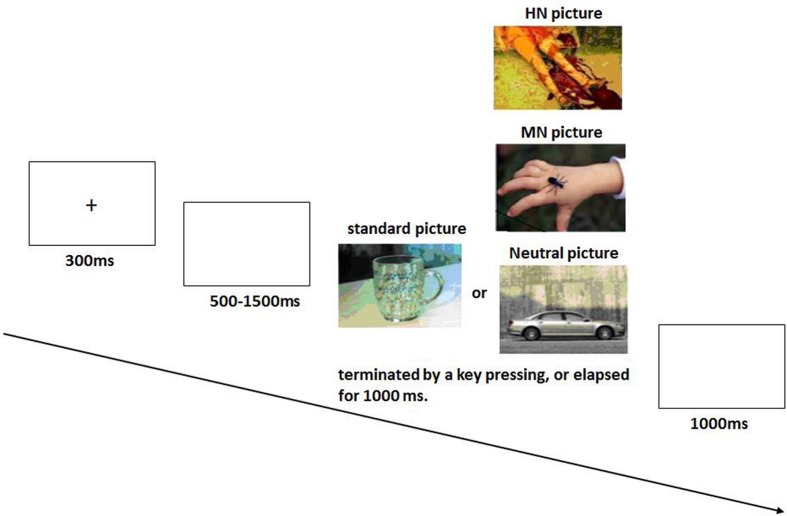
**The diagram of our experimental task (Yuan et al., 2015)**.

The present study performed a two-choice oddball task which consisted of 4 blocks of 100 trials, with each block including 70 standard and 30 deviant (grouped into three emotion conditions) pictures (**Figure 1**). All deviant pictures were taken from the Chinese adapted version of International Affective Picture System (Chinese Affective Picture System; Bai et al., 2005). A natural scene of a cup served as the frequent standard picture and 30 pictures grouped as either HN, MN, or Neutral served as the deviants. The pictures covered a variety of contents, such as highly unpleasant, mildly unpleasant, or neutral animals (e.g., snakes, bugs, or eagles), natural scenes (e.g., fire disaster, flood, clouds) and human activity (e.g., homicide, violence, or sports), but did not include single faces. The stimulus presentation was randomized across conditions. The validity of the three picture sets to serve as HN, MN, and neutral stimuli was verified by our prior study (Yuan et al., 2012). Contrast and luminance levels among photographs were also controlled. All the pictures were identical in size and resolution (15 cm × 10 cm, 100 pixels per inch).

### Procedure

The participants were seated in front of a monitor, about 150 cm from the screen, with the horizontal and vertical visual angles below 6°. To prevent fatigue, participants were allowed to take a short break for around 2 min after each block. Total testing time was approximately 35 min. Stimuli were presented using E-Prime version 1.1 (Psychological Software Tools, Pittsburgh, PA, USA). In each trial, a 300 ms fixation cross was presented, and was followed by the appearance of a black screen for 500–1500 ms. Then, a stimulus picture appeared on the screen. Subjects were instructed to press the “F” key on the keyboard with their left index finger as accurately and quickly as possible if the frequent standard picture appeared, and to press the “J” key with their right index finger if the infrequent picture appeared. The stimulus picture was terminated by a key pressing, or was terminated when it elapsed for 1000 ms. Therefore; each subject was informed that their responses must be made under 1000 ms. Each response was followed by 1000 ms of a blank screen. A practice session with 10 trials was used before the experiment in order to familiarize subjects with the procedure. The standard picture in the practice session was the same as that in the subsequent experiment whereas the deviants for practice were neutral pictures that were not selected for the formal experiment. All subjects achieved 100% accuracy in practice trials.

### Electrophysiological Recording and Data Analyses

The EEG was recorded from 64 scalp sites using tin electrodes mounted in an elastic cap (Brain Product, Munchen, Germany), with the average references on the left and right mastoids and a ground electrode on the medial frontal aspect (Luck, 2005). The vertical electrooculograms (EOGs) were recorded supra- and infra-orbitally at the left eye. The horizontal EOG was recorded from the left versus right orbital rim. The EEG and EOG were amplified using a DC~100 Hz bandpass and continuously sampled at 500 Hz/channel. All inter-electrode impedance was maintained below 5 kΩ. EEG epochs were segmented in 2200 ms time window with pre-stimulus 800 ms, and baseline corrected using 200 ms interval prior to picture onset. The epoched data were imported to EEGLAB toolbox^1^ (Delorme and Makeig, 2004) running under Matlab7.8.0 (MathWorks, Natick, MA, USA). The resulting complex signal provides an estimate of instantaneous power for each time point at frequencies of 3–100 Hz. This procedure is done on each trial, and then power values are averaged across trials. The data were highpass filtered at 0.05 Hz and average referenced across all scalp electrodes. Epochs with large artifacts (exceeding ± 100 μV) were removed. Independent component analysis (ICA) using the Infomax algorithm was used to obtain independent components (ICs) from scalp EEG activity and ICs represent artifacts were rejected.

**FIGURE 2:**
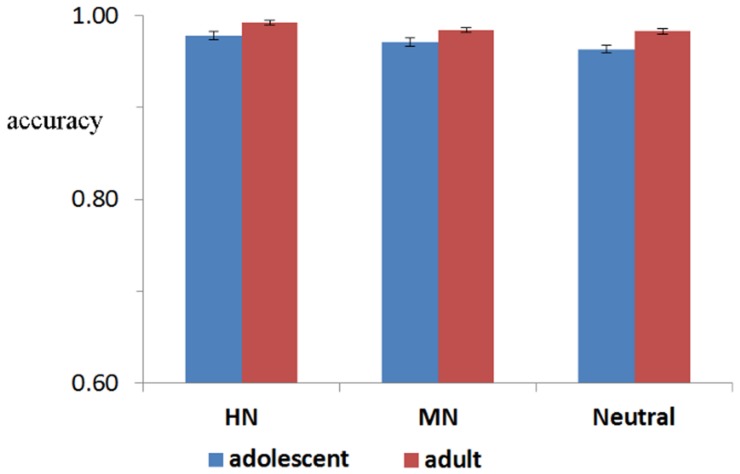
**The accuracy for adults and adolescents during HN, MN, and Neutral stimuli**.

**FIGURE 3:**
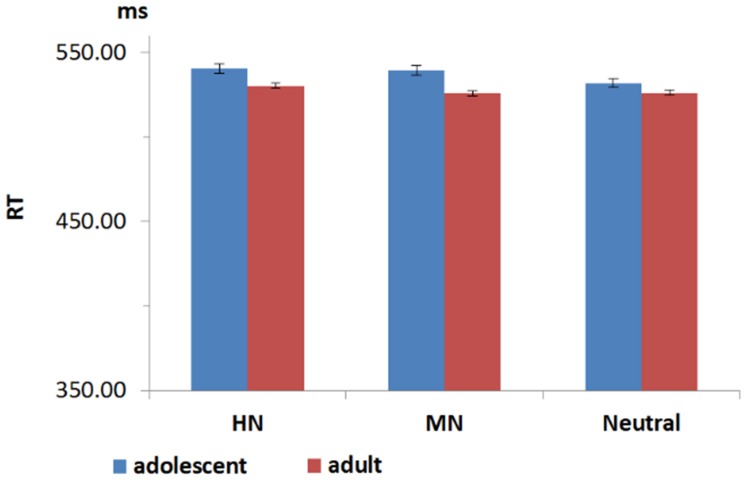
**The reaction time (RT) for adults and adolescents during HN, MN, and Neutral stimuli**.

**FIGURE 4:**
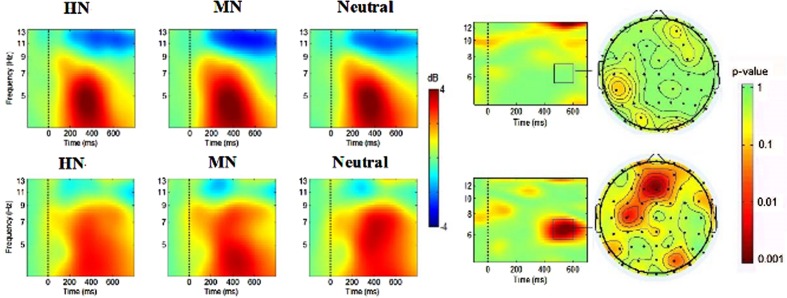
**(Left)** The averaged EEG oscillatory activities during HN, MN, and Neutral conditions at Fz over time (*x*-axis, 0 denotes picture onset) and frequency (*y*-axis, 3–12 Hz). Blue color indicates power decrease relative to pre-stimulus baseline and red color indicates power increase relative to the baseline. **(Right)** The time-frequency map and the scalp distribution of the emotion effect across HN and MN stimuli in 500–600 ms (**Right**, red color denotes smaller *p* values, and stronger significance level).

Trial-by-trial TFA was computed by a Morlet wavelet with linearly increased cycles, from 3 cycles at the lowest frequency (3 Hz) and 25 cycles at the highest frequency (125 Hz) analyzed. The frequency resolution = (125−3)/100 = 1.22. time resolution = 2000 ms/200 = 10 ms. Changes in event related spectral power response (in dB) were computed by the ERSP index (Makeig, 1993) (1),

$$\text{ERSP}\left(f,t\right)=\frac{1}{n}\overset{n}{\underset{k-1}{\Sigma }}{\left(F_{\text{k}}\left(f,t\right)\right)}^{2}$$

where, for n trials, *F*_k_(f, t) is the spectral estimate of trial k at frequency f and time *t*. Power values were normalized with respect to a 200 ms pre-stimulus baseline and transformed into decibel scale (10_log10 of the signal). A permutation test was conducted using the statcond function of the EEGLAB toolbox to identify the frequency band and time window that the ERSP values significantly distinguished between negative and neutral conditions. Based on the mass-univariate analysis, there was no significant emotion effect in the gamma (30–100 Hz) oscillation. However, the ERSP values within three time-frequency bands (theta: 5.5–7.5 Hz and beta: 18–20 Hz over 500–600 ms; alpha:10.5–12 Hz over 850–950 ms) showed significant differences between negative and neutral conditions (**Figures 2**, **3**, and **4**). Both theta and beta values were different for negative and neutral conditions across central and frontal scalp regions. Therefore, the ERSP data in the theta (5.5–7.5 Hz, 500–600 ms) and beta (18–20 Hz, 500–600 ms) bands were submitted into the ANOVA in the following 12 electrode sites: Frontal (F3, Fz, and F4), Frontal-central (FC3, FCz, and FC4), central (C3, Cz, and C4) and centroparietal (CP3, CPz, and CP4) sites. A repeated-measure ANOVA was performed with 3 (emotion: HN, MN, and Neutral)*4 (frontality: frontal, frontocentral, central, and centroparietal)*3 (laterality: left, midline, and right) *2 (group: adults, adolescents). Additionally, for the alpha frequency band, it has generally been found that emotional stimuli elicited emotional effect over parietal and/or occipital areas (Schmidt and Trainor, 2001; Simons et al., 2003; Balconi et al., 2009; De Cesarei and Codispoti, 2011; Cui et al., 2013). Therefore, the ERSP data in alpha time-frequency band was submitted into the ANOVA in the following 6 electrode sites: (PO3, PO4, POz, Oz, O1, and O2). The degrees of freedom of the F-ratio were corrected for violation of spherical assumption according to the Greenhouse–Geisser method. Bonferroni correction method was used for *post hoc* comparisons if significant main or interaction effects were found.

## Results

### Behavioral Result

Response accuracy (ACC) reached a ceiling effect, regardless of stimulus category and group manipulations (Macc = 98.5%). A repeated measures ANOVA of accuracy data with group (two levels: adolescents and adults) × Emotion (three levels: HN, MN, and Neutral) as factors yield neither significant main effects of group [*F*(1,38) = 1.487, *p* = 0.23] and emotion [*F*(2,76) = 2.459; *p* = 0.11], nor a significant group by emotion interaction [*F*(2,76) = 0.184; *p* = 0.23]. A repeated measures ANOVA of RT data yield neither significant main effects of group [*F*(1,38) = 0.08, *p* = 0.71] and emotion [*F*(2,76) = 1.60; *p* = 0.21], nor a significant group by emotion interaction [*F*(2,76) = 1.85; *p* = 0.16].

### EEG Result

#### Theta Analysis (5.5–7.5 Hz, 500–600 ms)

**FIGURE 5:**
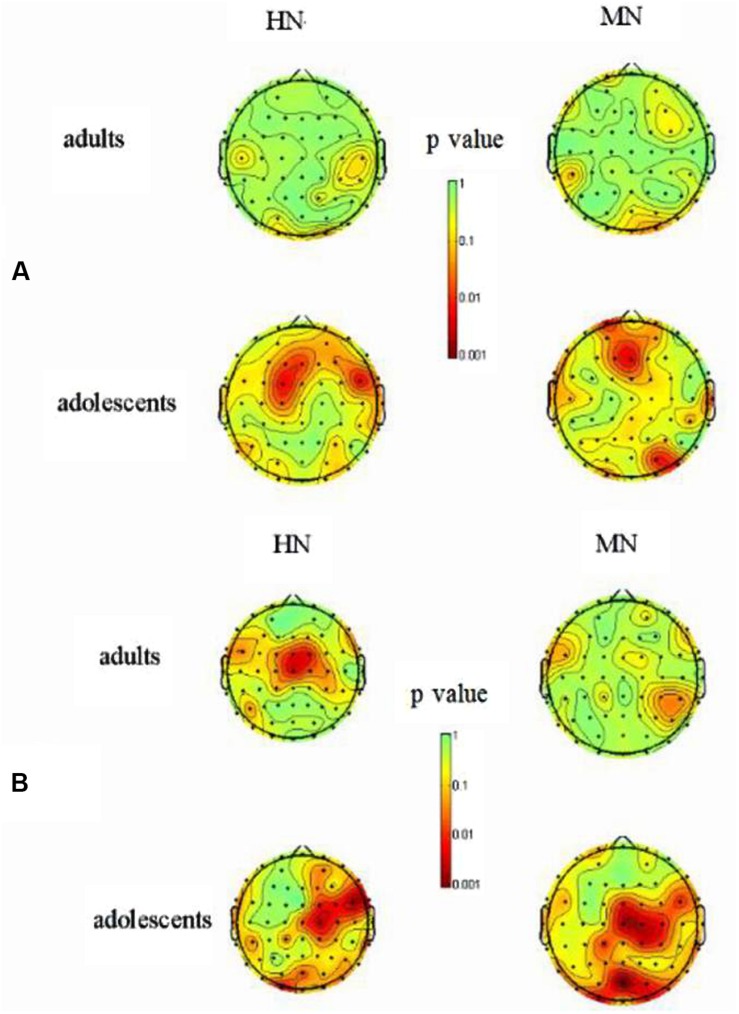
**(A)** The scalp distribution of the emotion effect on theta for HN and MN stimuli in 500–600 ms (red color denotes smaller *p* value and stronger significance level); **(B)** The scalp distribution of the emotion effect on beta for HN and MN stimuli in 500–600 ms (red color denotes smaller *p* value and stronger significance level).

There was a significant group by emotion interaction [*F*(2,76) = 3.16, *p* < 0.05] (**Figure 5**). The breakdown of this interaction showed a significant main effect of emotion in adolescents [*F*(2,38) = 4.15, *p* < 0.03] but not in adults [*F*(2,38) = 0.64, *p* = 0.713]. Adolescents were associated with significantly smaller theta ERS during HN [2.01dB; *F*(1,19) = 8.22, *p* < 0.02] than during Neutral conditions [2.44 dB; *F*(1,19) = 8.22, *p* < 0.02] while MN (2.13 dB) and Neutral conditions [2.44dB; *F*(1,19) = 2.975, *p* = 0.16] showed no significant differences. Additionally, the theta ERS is more pronounced at midline (2.05 dB) compared to left (1.52 dB) and right (1.05 dB) regions, indicated by a significant laterality effect [*F*(2,76) = 29.31, *p* < 0.001].

#### Beta Analysis (18–20 Hz, 500–600 ms)

**FIGURE 6:**
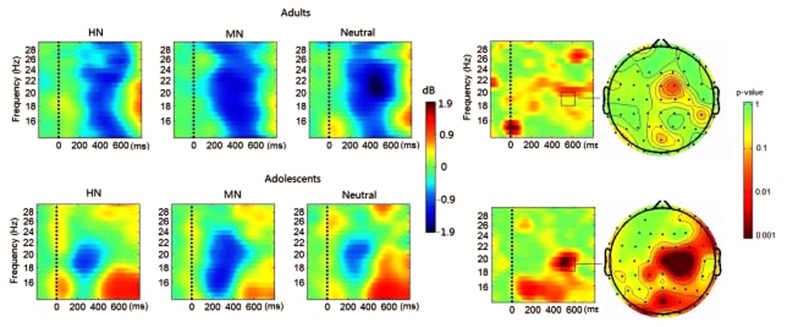
**(Left)** The averaged beta rhythm oscillations during HN, MN and Neutral conditions at Cz over time (*x*-axis, 0 denotes picture onset) and frequency (*y*-axis, 14–30 Hz). Blue color indicates power decrease relative to pre-stimulus baseline and red color indicates power increase relative to the baseline. **(Right)** The time-frequency map and the scalp distribution of the emotion effect across HN and MN stimuli in 500–600 ms (Right, red color denotes smaller *p* values and stronger significance level).

The analysis of beta activity showed a significant three-way interaction amongst emotion, group and frontality [*F*(6,228) = 2.93, *p* = 0.02]. The *post hoc* analysis showed a significant group by emotion interaction at frontocentral [*F*(2,76) = 4.67; *p* = 0.017], central [*F*(2,76) = 10.58; *p* = 0.001], and centroparietal regions [*F*(2,76) = 4.698; *p* = 0.013] but not in the frontal region [*F*(2,76) = 0.29, *p* = 0.741]. Therefore, we decomposed the group by emotion interaction, by testing the emotion effect in adults and adolescents, respectively, over frontocentral to centropareital regions. The emotion effect was significant in adolescents [*F*(2,38) = 8.91, *p* = 0.001] (**Figure 6**). Adolescents were associated with significantly larger beta ERD during HN [−0.578 dB; *F*(1,19) = 3.51, *p* = 0.004] and MN [−0.713 dB; *F*(1,19) = 3.00, *p* = 0.008] relative to Neutral (-0.09 dB) conditions. The beta ERD was similar across HN and MN conditions [*F*(1,19) = 0.83, *p* = 0.42]. By contrast, the emotion effect was non-significant in adults [*F*(2,38) = 1.73, *p* = 0.20]. Lastly, there was a significant frontality effect [*F*(3,114) = 40.26, *p* < 0.001], with the ERD most pronounced at central (-0.91 dB) and centroparietal (-1.05 dB) regions.

#### Alpha Analysis (10.5–12 Hz, 850–950 ms)

**FIGURE 7:**
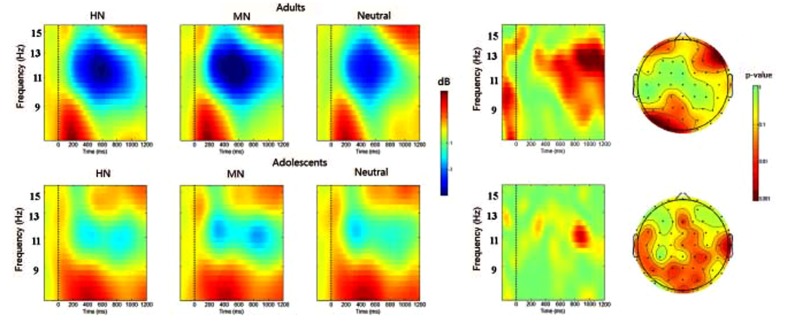
**(Left)** The averaged alpha rhythm oscillations during HN, MN, and Neutral conditions at Oz over time (*x*-axis, 0 denotes picture onset) and frequency (*y*-axis. 7–17 Hz). Blue color indicates power decrease relative to pre-stimulus baseline and red color indicates power increase relative to the baseline. **(Right)** The time-frequency map and the scalp distribution of the emotion effect across HN and MN stimuli in 850–950 ms window (Right, red color denotes smaller *p* values and stronger significance level).

The analysis of alpha activity showed a main effect of emotion [*F*(2,38) = 10.413, *p* < 0.001], while the emotion by group interaction was not significant [*F*(2,38) = 1.397, *p* = 0.253]. The *post hoc* comparisons with bonferroni correction showed larger alpha ERD during HN [−2.087 dB; *F*(1,19) = 8.883, *p* = 0.005] and MN [−2.488 dB; *F*(1,19) = 34.973, *p* < 0.001] relative to Neutral (-1.373 dB) conditions, irrespective of age. The alpha ERD was similar across HN and MN conditions [*F*(1,19) = 1.774, *p* = 0.191] (**Figure 7**).

## Discussion

We observed smaller theta ERS and larger beta ERD during HN compared to Neutral stimuli in adolescents, instead of adults. This suggests that the enhanced emotional response to HN stimuli in this time window is more robust, in adolescents relative to adults. It is worth noting that, a number of studies demonstrated that theta power is negatively related to anxiety (Knott et al., 1996; Mizuki et al., 1996; Suetsugi et al., 1998; Aftanas et al., 2003; Sachs et al., 2004; Knyazev et al., 2008). We observed smaller theta ERS during HN compared to Neutral stimuli in adolescents, instead of adults. This is likely because that compared to adults, adolescents are more sensitive to the HN stimuli and likely to show anxiety during HN stimuli than during neutral stimuli. However, the lack of direct measurement of anxiety when subjects were viewing negative events, was a weakness in the present study. Additionally, while MN stimuli elicited a significant emotion effects in beta ERD in adolescents, adults did not exhibit any emotion effect for MN stimuli in beta ERD. In addition, it has been considered that both theta ERS and beta ERD are related to orienting attention (Balconi and Lucchiari, 2006; Balconi and Pozzoli, 2009; Chen et al., 2015). These results suggest that adolescents are more sensitive to the negative scenes than adults, such that their attention could be directed to the content of the HN and MN stimuli despite engagement in a non-emotional distracting task. That is, it is harder for adolescents to ignore and distract attention away from emotional stimuli compared to adults. Previous studies using adult samples indicated that the attention orienting to negative stimuli was modulated by attention focus: while compared to neutral faces, fearful faces elicited enhanced neural response to emotional processing when attention was focused on faces (Holmes et al., 2003; Pourtois et al., 2004), this emotional effect was completely eliminated when attention was diverted away from the faces (Holmes et al., 2003). Furthermore, this attention orienting to negative stimuli was considered to be mediated by amygdala activity (Holmes et al., 2003; Rotshtein et al., 2010). It is worth noting that we used a non-emotional distracting task that diverts subjects’ attention away from the emotionality of pictures. This probably explains why we did not observe significant emotion effects in beta-band ERD and theta-band ERS in the adult sample. Nevertheless, adolescents showed significant emotion effects for both HN and MN stimuli, despite involvement in the distracting task. This explanation is consistent with our prior finding that the early attention effect for negative stimuli (e.g., in N1 and P2 components) was significant in adolescents but not in adults (Yuan et al., 2015). It was indicated that adolescence is associated with the immature prefrontal control system and the relative maturity of subcortical systems (Giedd et al., 1999; Sowell et al., 2002; Somerville et al., 2010). Consequently, the amygdala signaling should be stronger in response to salient stimuli in adolescents compared to adults, which may explain the pronounced emotional effect for both HN and MN stimuli in adolescents but not in adults.

Alpha band ERD has been reported to be involved in the regulation of negative emotion (Mu et al., 2008). The present study observed larger alpha ERD during HN and MN compared to Neutral stimuli, irrespective of adolescents or adults. This is consistent with previous finding that the alpha desynchronization was larger during negative relative to neutral stimulation (Schmidt and Trainor, 2001; Simons et al., 2003; Balconi et al., 2009; De Cesarei and Codispoti, 2011; Cui et al., 2013). These results suggest that adolescents and adults have similar emotional reactions in alpha oscillation to negative stimuli, regardless of valence strength. This may be due to the recruitment of a similar top–down controlled processing across samples, that is, similar inhibition of task-irrelevant emotional meanings to focus on the task of standard/deviant distinction (Yuan et al., 2012, 2015). This assumption was supported by our behavioral results demonstrating no significant age differences in both response time and accuracy.

Adults show a robust emotion effect for HN and MN stimuli only in alpha ERD. However, adolescents showed robust emotion effects in not only alpha ERD, but also theta ERS and beta ERD. These results suggest that when adolescents and adults recruit a similar top–down controlled processing, the regulation of attention orientation to negative stimuli may be more effective in adults compared to adolescents. Previous studies have indicated that there is a maturity imbalance between prefrontal cortex and subcortical limbic regions during adolescence: adolescence is characterized by immature prefrontal function and relative maturity of subcortical structures (Giedd et al., 1999; Sowell et al., 2002). This may enable stronger signaling of subcortical system (e.g., amygdala) paired with weaker top–down control signaling from neocortices in comparison with adults (Somerville et al., 2010). This probably explains the robust theta and beta emotion effects for negative stimuli in adolescents but not in adults, whose prefrontal regulation function has reach maturity (Somerville et al., 2010).

In summary, the current theta ERS and beta ERD provided converging evidence of enhanced brain susceptibility to negative stimuli in adolescents compared to adults. These results confirmed our prior findings (Yuan et al., 2015). Notably, as the present study only included negative stimuli, it is unclear whether the adolescent susceptibility to emotional stimuli found in the current study is dependent on stimulus valence (i.e., positive or negative). This issue is worthy of further investigation by comparing ERSP induced by negative and positive stimuli in future studies. The enhanced sensitivity to negative stimuli, and the developing prefrontal system may account for why adolescence is associated with the increased susceptibility to affective and behavioral disturbances, such as depression, anxiety disorders and aggression (Hankin et al., 1998; Rudolph, 2002).

## Author Contributions

XM and JY conducted the experiment and analyzed data. XM, WL, LZ, and JY proposed the concept of the measurements. BY and JY helped in the experimental design. XL and XM supervised the project. XM, XD, and JY conducted theoretical investigations leading to presented simulations. All authors discussed and contributed to the manuscript.

## Conflict of Interest Statement

The authors declare that the research was conducted in the absence of any commercial or financial relationships that could be construed as a potential conflict of interest.The reviewer KK and handling Editor declared their shared affiliation, and the handling Editor states that the process nevertheless met the standards of a fair and objective review.

## Footnotes

1 http://sccn.ucsd.edu/eeglab/
